# Mishearing as a Side Effect of Rational Language Comprehension in Noise

**DOI:** 10.3389/fpsyg.2021.679278

**Published:** 2021-09-06

**Authors:** Marjolein Van Os, Jutta Kray, Vera Demberg

**Affiliations:** ^1^Department of Language Science and Technology, Saarland University, Saarbrücken, Germany; ^2^Department of Psychology, Saarland University, Saarbrücken, Germany; ^3^Department of Computer Science, Saarland University, Saarbrücken, Germany

**Keywords:** speech comprehension, background noise, mishearing, false hearing, predictive context, aging

## Abstract

Language comprehension in noise can sometimes lead to mishearing, due to the noise disrupting the speech signal. Some of the difficulties in dealing with the noisy signal can be alleviated by drawing on the context – indeed, top-down predictability has shown to facilitate speech comprehension in noise. Previous studies have furthermore shown that strong reliance on the top-down predictions can lead to increased rates of mishearing, especially in older adults, which are attributed to general deficits in cognitive control in older adults. We here propose that the observed mishearing may be a simple consequence of rational language processing in noise. It should not be related to failure on the side of the older comprehenders, but instead would be predicted by rational processing accounts. To test this hypothesis, we extend earlier studies by running an online listening experiment with younger and older adults, carefully controlling the target and direct competitor in our stimuli. We show that mishearing is directly related to the perceptibility of the signal. We furthermore add an analysis of wrong responses, which shows that results are at odds with the idea that participants overly strongly rely on context in this task, as most false answers are indeed close to the speech signal, and not to the semantics of the context.

## Introduction

### Noisy Channel Model of Rational Communication

When listening to speech, there are usually at least two sources of information available to decode the speaker’s message: There is the sensory information in the form of the acoustic speech signal, and there is also contextual information that can help guide predictions (Boothroyd and Nittrouer, 1988; Nittrouer and Boothroyd, 1990). We rarely listen to other people speaking in perfectly quiet surroundings. Often, there is a lot of noise going on in the background, for example, other people speaking, traffic noise, or working machinery. The noise puts extra strain on our speech comprehension processes, something especially older adults can struggle with (Li et al., 2004).

Comprehenders take into account uncertainty in the perceptual input (for example, due to background noise). The noisy channel model (Shannon, 1949; Levy, 2008; Levy et al., 2009) proposes that language comprehension is a rational process, where we make use of all available sources of information. Bottom-up information from the speech signal is supplemented with top-down predictions of what the speaker is likely to say. Combining these two sources of information are a sensible strategy to maximize comprehension. Let’s take, for example, the sentence “He buys the bar,” In background noise, the listener might comprehend this as “He buys the car,” where *car* might be more probable given the context of buying than *bar*, while sounding similar. The actual comprehended word w’ is determined as w’=argmax_i_ P(w_i_|context) ^*^ P(s|w_i_) where P(w_i_|context) is the probability of the word given the preceding context, i.e., the top-down probability of a word, and P(s|w_i_) is the probability of the perceived signal for that word w_i_. The task of the listener consists of identifying candidate word w_i_ for which this probability given context and probability of signal fitting that word is maximal. This means that there is a trade-off between top-down and bottom-up information, where the probability distribution is shaped differently depending on the clarity of the acoustic signal. A noisier signal leads to a flatter distribution: There are more words w_i_ for which the perceived signal s has a relatively high probability, compared to a situation in which signal s is clearly intelligible. In cases where we therefore have a relatively flat probability distribution for P(s|w_i_), the top-down probability P(w_i_|context) will dominate what comes out as the most likely word w_i_ in the argmax calculation (besides words like *car*, also other words that frequently occur in a context of buying that share some overlap with the signal are probable based on the context). Under high noise, the top-down information will hence count more than the uncertain bottom-up information due to the stronger peaks in its distribution, leading to stronger reliance on prediction. In most cases, this will be beneficial to language comprehension, as it means that likely words can still be deciphered under noisy conditions. However, these predictions can also come at the cost of *mishearing*, where speech is misunderstood due to strong expectations (Rogers et al., 2012; Sommers et al., 2015; Failes et al., 2020). Rogers et al. (2012) explained this mishearing effect through general deficits in cognitive control for the older adults. They additionally report that older adults do not only show increased levels of mishearing compared to younger adults, but that they also report higher confidence in having heard a word which was not actually spoken.

In the present article, we argue that the larger mishearing effect observed in older adults compared to younger adults may be a simple consequence of rational integration of the bottom-up and top-down information, i.e., that their performance is not necessarily an effect related to deficits in cognitive control, but may reflect a combination of stronger top-down expectations due to increased linguistic experience, and lower confidence in the bottom-up input, due to first experiences of hearing loss. We have controlled our stimuli in such a way that in case of general cognitive causes, we should find no difference between our items (cognitive control should not be affected), while if the mishearing effect depends on clarity of the signal, we will find differences in comprehension performance. Different sound types have different signals that are easier or more difficult to distinguish in background noise, and the noisy channel model predicts that even minor changes in how well the acoustic signal can be perceived, can lead to a difference in the trade-off between top-down and bottom-up information. For example, the short burst of plosives is harder to distinguish in background noise than the steadier signal of a vowel. The noisy channel model would predict that in stimuli with plosives, listeners rely more on top-down prediction than in stimuli with vowel contrasts.

In the present study, we aim to investigate how background noise affects speech comprehension in younger and older adults, in situations where there is a predictive sentence context available that might facilitate or hinder speech recognition. Comparing younger and older adults is interesting, as older adults have more language experience and hence should have better expectations (Pichora-Fuller, 2008; Sheldon et al., 2008), while at the same time, they may already be subject to some hearing loss and know to trust the incoming signal less. Given both of these factors, we would predict based on the noisy channel model that older adults show larger effects of the top-down predictions on interpretation, and thus be subject to stronger mishearing effects than younger adults.

In the following sections, we will describe the effect of background noise on speech understanding in general (“Effect of Background Noise on Speech Understanding”), and in older adults more specifically (“Age Differences in Language Comprehension Under Noise”). We will discuss false hearing in more detail (“Age-Related Differences in False Hearing”) and introduce the aims of the current study (“The Present Study”).

### Effect of Background Noise on Speech Understanding

Background noise has a negative effect on speech comprehension in younger as well as in older adults. It can lead to energetic masking, where both the speech signal and the competing noise have energy in the same frequency bands at the same time (Brungart, 2001). The acoustic cues that listeners need for sound identification are masked by the noise, or if the background noise is competing speech, its acoustic cues can “attach” themselves to the target speech (Cooke, 2009). The type of noise, for example, white noise, babble noise, or competing speech from a single speaker, might have different effects on the target speech. The present study uses multi-speaker babble noise, where none of the speakers are understandable.

Relevant for the current study is also the distinction that can be made between consonants, in particular plosives, and vowels. These different types of sound might be affected in different ways by various types of background noise. Plosives, on the one hand, consist of a closure of some part of the vocal tract, followed by a short burst of energy. This burst can easily be masked by noise, if that happened at the same time. On the other hand, vowels generally have a longer, more steady signal with a higher intensity, that can be easier to distinguish in background noise. Their energy primarily lies between 250 and 2000Hz (first and second formant, Flanagan, 1955), thus lower than that of consonants, which have information also in higher formants (Edwards, 1981; Alwan et al., 2011). Spectral frequency information is in particular important for identifying the place of articulation in plosives (Liberman et al., 1954; Edwards, 1981).

When it comes to background noise, not only the type of background noise matters, but also the level of the noise, the level of background noise is commonly measured in Signal to Noise Ratio (SNR). It quantifies the relation between the amplitude of the speech signal and the amplitude of the background noise. A negative SNR means that the background noise is stronger than the speech signal (which is thus more difficult to understand), and a positive SNR means that the speech signal is stronger than the background noise. In the case of 0 SNR, both the noise and the speech are equally strong. In the present study, the noise levels have been set at 0 SNR and −5 SNR, so that we can investigate whether mishearings change as a function of the difficulty of the listening condition.

### Age Differences in Language Comprehension Under Noise

There are differences between younger and older adults even in quiet situations. With increasing age, there are changes in auditory processing (Gordon-Salant et al., 2010; Helfer et al., 2020). In particular, changes in the inner ear and neural pathways can lead to age-related hearing loss, presbycusis, in which the highest frequencies (4–8kHz) are most affected and continue to get worse in older adults (Gates and Mills, 2005). When the hearing loss progresses to frequencies of 2–4kHz, this affects speech comprehension, and in particular understanding of voiceless consonants. Older adults also often have reduced ability to differentiate between different frequencies, to discriminate spectral and temporal transitions in the speech signal, and to localize sound sources (Schuknecht and Gacek, 1993; Chisolm et al., 2003; Tun et al., 2012; Helfer et al., 2020). These declines lead to greater difficulty understanding speech in adverse listening conditions (Pichora-Fuller et al., 1995; Li et al., 2004; Schneider et al., 2005; Pichora-Fuller et al., 2017). Additionally, there are cognitive changes with increasing age. Older adults have been found to show decreased attention, working memory, executive functions, and processing speed (Salthouse, 1990, 1996; Lindenberger and Ghisletta, 2009; Tun et al., 2012; Tucker-Drob et al., 2019). These abilities all play a role in speech comprehension, which will thus be negatively impacted as well.

General language abilities are well preserved in old age, and older adults are able to compensate for their reduced auditory and cognitive abilities by using knowledge-based factors, such as supportive sentence context (Stine and Wingfield, 1994; Wingfield et al., 1995, 2005). Studies compared groups of younger adults with groups of older adults to determine how noisy environments and informative contexts might affect the latter group differently than the former (Hutchinson, 1989; Pichora-Fuller et al., 1995; Sommers and Danielson, 1999; Dubno et al., 2000; Benichov et al., 2012). The results showed that older adults are generally more adversely affected by background noise than younger adults and that older adults rely more heavily on the provided sentence context than younger adults. In fact, older adults have been shown to rely on contextual prediction to such an extent that the predictions can make up for the adverse effect of noise (Wingfield et al., 2005) and other adverse listening conditions (Wingfield et al., 1995; Lash et al., 2013). Older adults might be particularly adept at using contextual information as a compensation mechanism, because every day they are exposed to challenging listening situations. They may have come to rely on using contextual cues to support speech comprehension processes, so that with age and experience, increased attention is allocated to higher-order knowledge structures (Steen-Baker et al., 2017). Koeritzer et al. (2018) investigated how background noise and ambiguous words in sentences affect recognition memory for spoken sentences. They presented the sentences in SNRs of +5 and +15, thus with an increased acoustic challenge, but with intelligible speech. Results showed that recognition memory was worse for acoustically challenging sentences and sentences containing ambiguous words, and older adults performed worse than younger adults in the ambiguous sentences in noise. Koeritzer et al. concluded that in particular older listeners rely on domain-general cognitive processes in challenging listening conditions, even when the speech is highly intelligible. Rogers et al. (2012) concluded that older adults are more biased to respond consistently with the context than younger adults, due to general deficits in cognitive control. However, other studies have argued that older adults’ reliance on context is due to predictions and more language experience (Wingfield et al., 2005; Sheldon et al., 2008).

### Age-Related Differences in False Hearing

Predictions made based on context might come at a cost. Older adults have been found to show higher rates of “false hearing” than younger adults (Rogers et al., 2012, p. 33). Here, false hearing is defined as a “mistaken high confidence in the accuracy of perception when a spoken word has been misperceived”. In their study, Rogers and colleagues used a priming paradigm in which they paired semantically related words (*barn*/*hay*). In a training phase, participants were familiarized with these associations. In a subsequent testing phase, the cue word (*barn*) was presented in clear listening conditions, and subsequently the target word was presented in noise. There were three conditions: (1) congruent, where the target word was the same as in the training phase (e.g., *hay*); (2) incongruent, where the target word was a phonological neighbor that formed a minimal pair with the word in training (e.g., *pay*); and (3) baseline, where the target word was unrelated to the training word (e.g., *fun*). Both younger and older adults indicated which words they had heard and how confident they were that they had identified the word correctly. The results of the study showed that older adults made use of the trained context more often and with more confidence than younger adults, even when the presented words were not matched in the training phase. Thus, older adults showed a larger false hearing effect than the younger adults. Comparable results using a similar priming paradigm have been found by Rogers and Wingfield (2015) and Rogers (2017). In a follow-up study, Rogers (2017) investigated whether the false hearing effect is caused by semantic priming or repetition priming, by manipulating the number of exposures to the training cue-target pairs. The results showed that an increased number of exposures did not increase the effect of false hearing, but that this effect was strongest when the cue-target pair was not presented at all during the training phase. These observations indicate that the false hearing effect is caused by semantic priming rather than repetition priming, suggesting that false hearing relies on top-down semantic associations in the context.

More recent studies have investigated false hearing using a more naturalistic paradigm than the priming paradigm used in previous studies. Sommers et al. (2015) and Failes et al. (2020) used sentences rather than word pairs, in three conditions. A neutral carrier phrase formed the baseline condition, and there were congruent (e.g., “The shepherd watched his sheep.”) and incongruent (“The shepherd watched his sheath.”) sentences. Here, the sentence-final target items differed in the first or last phoneme, while controlling for frequency and neighborhood density. Participants listened to the sentence in quiet, and the target item embedded in babble noise. Identification accuracy and confidence ratings were analyzed, showing that older adults performed better than younger adults on congruent trials, but had a higher false alarm rate for the incongruent trials. Older adults were more confident of these false alarms than younger adults, showing the increased false hearing effect for older participants.

Like these two previous studies, the present study investigated the predictability of the target word based on the context, but in German instead of English. While we are mainly interested in mishearings, we do collect confidence ratings of the participants’ responses to also investigate false hearing. Unlike previous studies, we systematically vary the sound type change between the target and distractor item so that only one phonetic aspect of the phoneme is changed, in order to investigate whether different types of sounds are affected by false hearing to a similar extent. Finding any differences between sound types (vowel quality vs. place of articulation in plosives) can help distinguish between accounts explaining the mishearing and false hearing effects, as this would mean listeners behave optimally based on the perceived information. If mishearing and false hearing in older adults is based on general deficits in cognitive control (Rogers et al., 2012), we should find the same effect for the different sound types.

Besides false hearing, larger effects for older adults compared to younger adults have been found for false memories (Hay and Jacoby, 1999) and false seeing (Jacoby et al., 2012). These processes seem to share a common mechanism, as Failes et al. (2020) found that participants who showed more false hearing, also were more likely to have false memories, and Jacoby et al. (2012) link false seeing to false hearing. In all cases, there seem to be top-down processes that lead to the false perceptions by overriding bottom-up signals (Bruner, 1957; Balcetis and Dunning, 2010 for false seeing; Roediger and McDermott, 1995 for false memory).

### The Present Study

Our study investigates how bottom-up auditory processes and top-down predictive processes interact in speech comprehension. We tested both younger and older adults in our experiment, as we expect age differences in the quality of top-down and bottom-up processes. Participants completed a word recognition task, where sentences were either presented in quiet or in background noise, and where the sentence context could be used to predict the sentence-final target word or not. These sentence-final target words were designed to be minimal pairs with respect to pronunciation, so that in the low predictability context, the word sounded very similar to the word that in fact did fit the sentence semantically. This allowed us to investigate whether listeners are able to rely on small acoustic cues for word recognition, even in background noise, while keeping sentence contexts equal across conditions.

The main question that the present study aims to address is the replicability of mishearings in German. Like previous studies (Sommers et al., 2015; Failes et al., 2020), we use a paradigm of word recognition in sentences, where the context is predictive or unpredictive of the target word. We add a quiet condition without added background noise as a baseline condition, which will allow us to make sure that hearing ability between groups is comparable with respect to our materials. It is also possible that we will observe a general increase of mishearing in older adults compared to younger adults, even in the quiet condition. This would be an interesting finding, as it would show that older adults rely more on context than the acoustic signal even if the acoustic signal is easily accessible, comparable to the finding that older adults rely more on domain-general cognitive processes in challenging listening conditions with high intelligibility (Koeritzer et al., 2018). Like previous studies, we will collect confidence ratings to investigate false hearing as a second point of interest.

To be able to distinguish between different accounts that explain the mishearing effect, we investigate the effect of noise on different types of speech sounds, and how these are affected by false hearing. We constructed our stimuli such that the minimal pairs in our experiments differed in just one feature: either vowel quality or place of articulation in plosives. The acoustic properties of our manipulation in vowels vs. plosives differ in various ways. First, vowel sounds have a longer and steadier signal compared to the relatively short burst of the plosives. Second, higher frequencies are more informative for plosives than for vowels, in particular for place of articulation (Liberman et al., 1954; Edwards, 1981; Alwan et al., 2011), which is the contrast in our minimal pairs. Based on the noisy channel model, we expected to find that the top-down predictions play a larger role in the case of plosives, as here the signal of the target and distractor are more similar to each other compared to the vowel condition, and thus will have more flat probability distributions (where both the target and the distractor have a similar probability of leading to the observed acoustic signal) based on the bottom-up processes. Listeners try to overcome this by relying more on the contextual information that is more easily accessible and gives distinguishing information. Furthermore, we expected that this difference between vowels and plosives may also be more pronounced in older adults, as hearing ability in high-frequency ranges is known to degrade during aging (Gates and Mills, 2005). Listeners optimally combine bottom-up and top-down probabilities, leading to mishearing in difficult listening conditions where the choice of the most likely word is mostly determined by the top-down prediction, an effect that is stronger for older adults as they compensate for age-related reductions in auditory and cognitive processing, but still rationally combine the acoustic and top-down information that is available to them.

## Materials and Methods

### Participants

A total of 93 native German speakers participated in the present experiment, for which we used the recruitment platform Prolific (prolific.co). We excluded seven older participants based on their performance in the quiet condition, because their number of distractor responses exceeded that of the younger adults. In this way, we ensured equal hearing abilities with respect to our stimuli across ages, as we were not able to collect hearing thresholds for our participants (because in-lab experiments were not possible at the time of conducting this study). The high number of unexpected responses in this relatively easy condition without background noise might also have been due to difficulty playing the audio or doing the task. The mean age of our final group of participants was 40years (age range=18–68years), 43 were male. While all participants were self-reported native speakers of German, their current countries of residence varied as: 55 lived in Germany, 12 in the United Kingdom, 4 in Austria, 3 in Ireland and Spain, 2 in the United States, 1 in each of France, Israel, Portugal, Poland, and South Korea. Three did not list their country of residence. Three out of our 87 participants reported to not speak other languages besides German, all three were older adults. From the remaining 84 participants, the languages spoken besides German were most often English (reported by 82 participants), French (reported by 21), and Spanish (reported by 14). In the post-experimental questionnaire, most participants reported no hearing issues or use of hearing aids. One participant (age 29) reported tinnitus, and one reported reduced hearing in his right ear (age 48, 60% hearing left). In order to check for any effects of education, we computed Spearman’s correlation between participants’ age and education level. This correlation was small (*ρ*=0.2, *p*=0.08), indicating that the older participants in our study were slightly more highly educated than the young participants. All participants gave informed consent, and the study was approved by the Deutsche Gesellschaft für Sprachwissenschaft Ethics Committee. The experiment lasted approximately 20 min and all participants received 3.12 Euro as compensation for their participation.

### Materials and Task

German minimal pairs were selected from the CELEX lexical database (Baayen et al., 1995), based on their phonetic transcription. These minimal pairs were chosen so that the contrast was in the middle of the word, rather than word-initial or word-final, as there were most pairs available for this position for the sound contrasts. In order to test the hypothesis that the effect of noise may be more detrimental to understanding the spoken target word for pairs that differed in a plosive than for those that differed in a vowel, we included both vowel contrasts (tense/lax: i/ɪ, y/ʏ, u/ʊ, ɛ/ɶ, o/ɔ, ɐ/ə) and plosive contrasts (paired on place of articulation contrasts: p/t, p/k, t/k, b/d, b/g, d/g). First, all pairs were inspected, and we excluded those that were not true minimal pairs (usual pronunciation differs from transcription), that had one or two too infrequent words (regionally used or technical terms), or those of which the words differed in gender or part of speech so that constructing stimuli for them was not possible. By controlling the phonetic contrast and part of speech of the words, we were not able to control for word frequency or neighborhood effects. Sentences were constructed around the minimal pairs, so that the target word appeared in sentence-final position and the word would be predictable from the sentence context. All stimuli were subjected to cloze testing using native German speakers on the Prolific platform. Cloze probabilities for each item were calculated based on the answers of 10 participants. We aimed for high cloze probabilities. Therefore, all stimuli that were still scoring too low on cloze probability were revised. Three rounds of cloze testing were completed, until we had 120 high-predictability sentence pairs (240 items in total). The cloze values ranged from 0.5 to 1 (mean=0.72) for the 136 items constructed under strict conditions. In 104 cases, the cloze was still quite low. We relaxed the high cloze requirement when even after multiple revisions, there was a high cloze competitor that differed only in the prefix (*laden* vs. *aufladen* for “to charge”*)* or that was too highly frequent to allow us to improve the sentence (*sieden* vs. more frequent *kochen* for “to boil”), and included these items even though they had a lower cloze probability than 0.5. The average cloze for all items, including those with the relaxed requirements, was 0.52. None of the participants took part in more than one of the rounds of cloze testing, and none of them participated in the main experiment.

To make the unpredictable stimuli, we swapped the two sentence-final target words, aiming for unpredictable but grammatically correct swaps wherever possible. In practice, this meant that all swapped sentences were unpredictable and implausible. Almost all sentences were still grammatically correct after swapping the target word, but two out of 240 swapped sentences became grammatically incorrect (for example, an argument was missing for a transitive verb). This resulted in 120 sets of four sentences, with two predictable and two unpredictable sentences of the minimal pair (*N*=480). Plausibility ratings were collected for all 480 items, again using the Prolific environment. Each item was rated 10 times, and ratings were averaged. Again, none of the participants took part in the main experiment. Plausibility was rated on a scale from 1 (completely implausible) to 5 (completely plausible). The predictable sentences had a mean plausibility rating of 4.60 (*SD*=0.41), and the unpredictable sentences had a mean plausibility rating of 1.73 (*SD*=0.59). Example stimuli can be found in Table 1.

**Table 1:** Example Stimuli.

1A	Am Pool im Hotel gab es nur noch eine freie **Liege**	HP
	*At the pool in the hotel there was only one free **lounger** left*	
1B	Nach vier Jahren heiratete Paul seine große **Liebe**	HP
	*After four years, Paul married his big **love***	
1C	Am Pool im Hotel gab es nur noch eine freie **Liebe**	LP
	*At the pool in the hotel there was only one free **love** left*	
1D	Nach vier Jahren heiratete Paul seine große **Liege**	LP
	*After four years, Paul married his big **lounger***	

Highly predictable sentences (HP) were made based on minimal pairs (Liebe/Liege) in 1A and 1B; then, sentence-final target words were swapped to make low-predictability items (LP) with the sentence frames of 1A and 1B, resulting in 1C and 1D. English translations have been given in italics.

Recordings were made of all predictable sentences (240 in total). The sentences were read by a female speaker, who was a native speaker of German. The speaker was instructed to read slowly and to pay attention to not include any slips of the tongue or hesitations. Sentences that were not read as intended or included slips of the tongue were repeated until each sentence was recorded in a clean version suitable for testing.

Unpredictable sentences were constructed via cross-splicing of the recordings of predictable sentences, in order to make sure that the intonation and stress patterns were identical across conditions and not indicative of the unpredictable items. The splicing was performed using Praat (Boersma and Weenink, 2020, version 6.1.05) and resulted in the total of 480 sentences. All cross-spliced unpredictable items were listened to carefully, to identify any problems related to cross-splicing, and corrected by adapting the slicing boundary or adapting the pitch contours. This was done by the first author as well as two native German student assistants. The final 480 sentences all sounded natural for the purposes of the experiment.

All sentences were embedded in background noise, which was café noise (BBC Sound Effects Library, Crowds: Interior, Dinner-Dance^1^), a multi-speaker babble noise where none of the speakers was intelligible. This was done in two different Signal to Noise (SNR) ratios, namely, 0 (meaning the target sound and the background noise were equally loud) and −5 (meaning that the background noise was 5 dB louder than the target sound). These values were chosen by the authors as challenging but not impossible to understand. As we are interested in the effect of background noise and sentence context on the intelligibility of the sentence-final target word, we took the mean intensity of each target word and calculated the SNR values based on this value, rather than the mean intensity of the sentence. Because the intensity of a spoken sentence tends to drop toward the end (Vaissière, 1983), it would mean the SNRs were actually lower for the target word, in case the mean sentence intensity was to be used. The noise was the same level throughout the sentence and started 300ms before sentence-onset and continued for 300ms after sentence-offset. This way, we gave participants a chance to focus on the speech in the noise. This was also the reason not to keep the sentence clear and embed only the target word in noise: We feared participants would not have time to get used to the added noise and it would be a less natural way of presenting the stimuli. Besides the noise conditions, there was a quiet condition, resulting in three different noise levels, quiet, 0 SNR, and −5 SRN.

### Design

The experimental items were arranged in a Latin Square design. Twenty-four different lists were constructed, consisting of 60 items each. These lists were constructed in such a way that each noise level and each predictability level occurred the same number of times and that each item appeared only once per list (same target pair or same predictability sentence). This was done in a crossed design, so that out of the 60 items, 30 were predictable and 30 were unpredictable. Out of each set of 30 items, 10 were presented in quiet, 10 in 0 SNR noise, and the remaining 10 in −5 SNR noise. The items were blocked by noise level, starting with 0 SNR, followed by −5 SNR, and ending with the quiet condition. This blocking was chosen to give participants a chance to maximally adapt to the noise and the task, starting with the relatively easy noise condition before being presented with the relatively hard noise condition. The quiet condition was presented at the end, so as not to give away the goal of the experiment at the start. Each list was preceded by a practice block, consisting of four items. This short practice block made the participants familiar with the task and online testing environment. All noise levels (quiet, 0SNR, and −5 SNR) were presented during the practice block.

### Procedure

**Figure 1:**
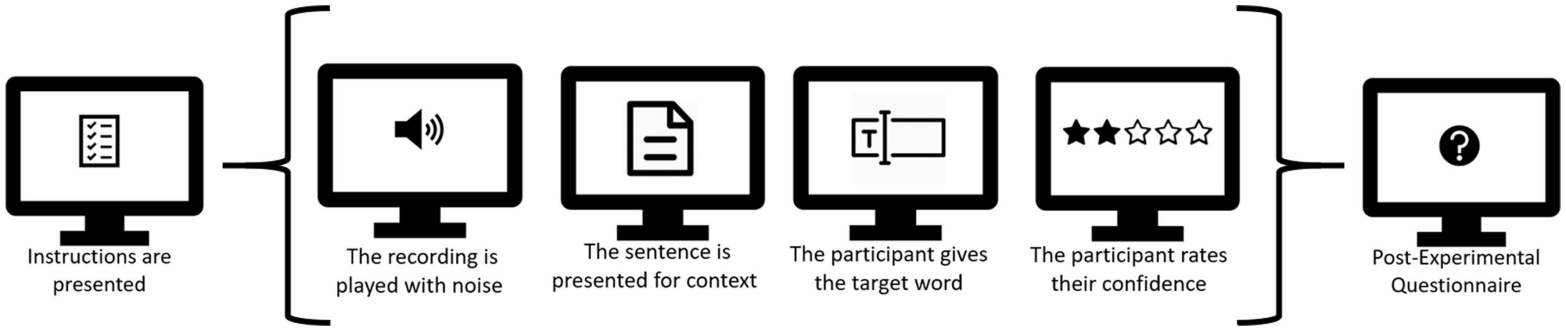
This figure shows the different stages of the experiment, with a single trial between brackets. Participants completed four practice trials and sixty experimental trials.

The experiment was hosted on Lingoturk, a crowdsourcing client (Pusse et al., 2016). Participants completed the experiment on a computer in a quiet room and using the Chrome web browser. They were instructed to use either headphones or speakers. In the experiment, participants had to listen to the sentence and report the final word they had heard. Before the start of the main experiment, the participant saw a series of instructions detailing the task. Participants were asked to listen carefully and report what they heard. We did not explicitly state that the sentences could be misleading. These screens included a sound check as well, so that the participant had the opportunity to make sure sound was being played correctly. In the main task of the experiment, the sentence, minus the target word, was presented on the screen in written form. We opted to include the written sentence up until the target word to make sure participants were able to use the context also in noisy conditions. A text box was provided for the participant to type their answer. Additionally, they rated their confidence in having given the correct answer on a scale from 1 (completely uncertain, guessed) to 4 (completely certain). At the start of a trial, the sound played automatically while the screen showed a fixation cross. Next, a screen with the two questions appeared after the recording had finished playing. The next item started playing as soon as the participant clicked to go to the next trial. As mentioned above, the experiment started with a short practice session consisting of four items, which were presented after the participant had seen all instructions. A schematic overview of the experiment is presented in Figure 1.

### Analysis

After data collection had been completed, all received answers were first classified automatically on whether it was the *target*, the word that was played in the audio (e.g., in example 1A in Table 1 “Liege”/“lounger”), the similar sounding *distractor* (e.g., in 1A “Liebe”/“love”), or were a different word entirely (e.g., in 1A “Platz”/“space,” *wrong*). The list of answers that had been classified as *wrong* was then checked by the first author and a native German-speaking student assistant, to correct misclassifications because of typos. In our statistical analyses, we included the trial number of each block as a control variable to check for any learning effects. We analyzed the high-predictability and low-predictability items separately due to ceiling effects in the high-predictability condition. To determine whether participants relied on the sentence context or on the speech signal, we coded the semantic fit of the incorrect responses (fitting or not fitting), as well as the phonetic distance between the incorrect responses and target and distractor items. We made phonetic transcriptions based on the Deutsches Aussprachewörterbuch (German Pronunciation Dictionary; Krech et al., 2009) and calculated the weighted feature edit distance using the Python package *Panphon* (Mortensen et al., 2016). This distance was normalized by dividing it by the longest of the two compared words. The normalized distance fell between 0 and 1.

## Results

In the first part of the result section, we will report the results on age differences in response accuracy in the high- and low-predictability conditions investigating mishearing. In the second part, we will analyze confidence ratings and investigate age differences in the false hearing effect. We used general linear mixed models (GLMM; Quené and Van den Bergh, 2008, for a tutorial see Winter, 2019), implemented in the lme4 package (Bates et al., 2015) in R (R Development Core Team, 2020) to analyze our data. These models allow both fixed and random effects, letting us control for variation on the participant- and item-level (Baayen et al., 2008; Barr et al., 2013). To improve convergence, all models were run using the bobyqa optimizer and increased iterations to 2·10^5^. Model comparisons were made to guide model selection based on the Akaike Information Criterion (AIC), and models with the lowest AIC are reported below.

### High Predictability Helps Comprehension in Noise

**Figure 2:**
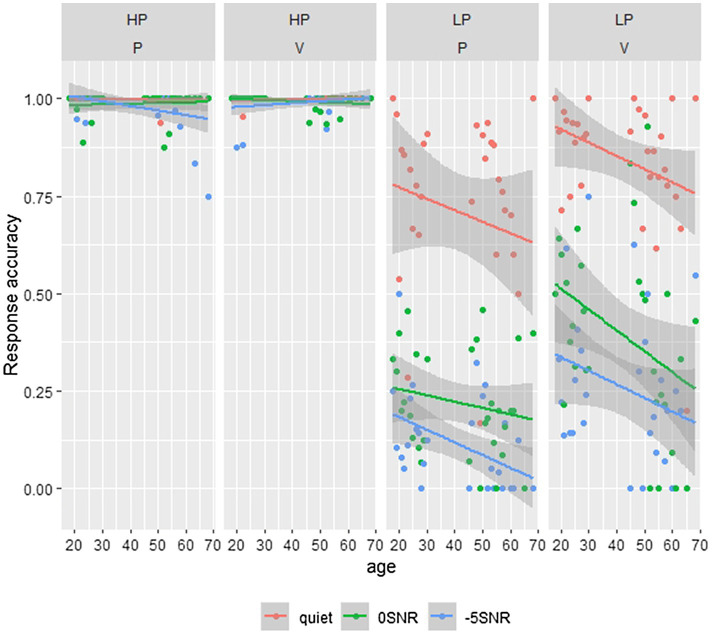
This figure shows the participant’s answers; split for target and distractor items, with age plotted on the x-axis and answer type on the y-axis. Here 0 denotes the distractor response and 1 the target response. Different line colors show different noise conditions. The different plots show the high (HP)- and low-predictability (LP) items for stimuli differing in a plosive (P) or vowel (V).

We are interested in whether listeners are able to pick up on small acoustic cues identifying words in minimal pairs, in quiet but also in background noise. For the initial analyses, we used a subset of our data consisting of the participants’ target and distractor answers, thus disregarding the wrong responses. We tested the participants’ binomial responses (0=distractor and 1=target) using a GLMM with a logistic linking function. First, we analyze the subset of the high-predictability items. In this analysis, all confidence ratings are collapsed. The model included fixed effects of Noise (categorical predictor with three levels using dummy coding and mapping the quiet condition to the intercept), Age (continuous predictor and scaled to improve convergence), ContrastVP (categorical predictor with two levels using dummy coding and mapping plosive to the intercept), and Trial Number (continuous predictor with trial number within each block and scaled to improve convergence). Additionally, the model included the interaction of ContrastVP and Age (scaled) and the interaction of Trial Number and Age (both scaled). The model included no random effects, since this led to non-converging models or singular fit. The model revealed a significant effect of Noise, where participants more often give the distractor answer noise compared to the quiet condition (*β*=−7.12, *SE*=1.70, *z*=−4.18, *p*<0.001 for 0 SNR and *β*=−6.27, *SE*=1.78, *z*=−3.55, *p*<0.001 for −5SNR). As can be seen in Table 2, all other effects were not significant (all *value of p*s>0.35). The noise effects are relatively small, and overall participants score close to ceiling, where most responses are target responses. These effects can also be seen in the two left-hand panels in Figure 2.

**Table 2:** Model outcomes for high- and low-predictability items.

	High-predictability items subset					Low-predictability items subset				
	Estimate	*SE*	*z*-value	*value of p*		Estimate	*SE*	*z*-value	*value of p*	
Intercept (quiet, P)	5.77	0.61	9.53	< 0.001	^***^	2.16	0.31	7.03	< 0.001	^***^
Noise −5SNR	−1.61	0.64	−2.48	< 0.05	^*^	−6.32	0.39	−16.09	< 0.001	^***^
Noise 0SNR	−1.38	0.65	−2.11	< 0.05	^*^	−4.87	0.32	−15.22	< 0.001	^***^
Age	−0.25	0.27	−0.93	0.35		−0.25	0.21	−1.18	0.24	
Trial No	−0.12	0.20	−0.61	0.54		0.47	0.08	5.97	< 0.001	^***^
ContrastVP V	0.24	0.40	0.61	0.54		1.55	0.34	4.63	< 0.001	^***^
Age: ContrastVP V	0.23	0.40	0.57	0.57		−0.33	0.14	−2.29	< 0.05	^*^
Age: Trial No	0.10	0.20	0.47	0.64		−0.004	0.08	−0.06	0.95

This table presents the analyses for the subsets of high and low predictability items. The response variable is the participants’ answer type, distractor (0), or target (1).

### Effects of Noise and Phoneme Change on Comprehension

The model for the low-predictability subset of the data included the same fixed effects as the high-predictability subset but included by-Participant and by-Item random intercepts and a random slope for Noise for the by-Item random intercept. The model revealed a significant effect of Noise, where the noise conditions had more distractor responses than Quiet (*β*=−4.87, *SE*=0.32, *z*=−15.22, *p*<0.001 for 0SNR and *β*=−6.32, *SE*=0.39, *z*=−16.09, *p*<0.001 for −5SNR). Additionally, the model revealed a significant effect of Trial Number (*β*=0.47, *SE*=0.08, *z*=5.97, *p*<0.001), meaning that participants slightly increased the amount of target responses with practice. The interaction of Age and Trial Number was not significant (*p*=0.95), suggesting older adults also showed this learning effect. The model also revealed that items of minimal pairs differing in the vowel had more target responses than items of minimal pairs differing in the plosive (*β*=1.55, *SE*=0.34, *z*=4.63, *p*<0.001). This was in line with the expectation that words differing in the plosive contrast would be harder to identify correctly than words differing in the vowel. Finally, the interaction of ContrastVP and Age was significant as well (*β*=−0.33, *SE*=0.14, *z*=−2.29, *p*<0.01), showing that with increasing age, there was a larger decrease in the proportion of target responses for vowel contrasts than plosive contrasts. These effects in general are presented in Table 2 and illustrated in the two right-hand panels of Figure 2. The interaction effect in particular is shown by the steeper downward slope of the lines in the LP vowel plot compared to the LP plosive plot.

### Semantic Fit and Phonetic Distance

**Figure 3:**
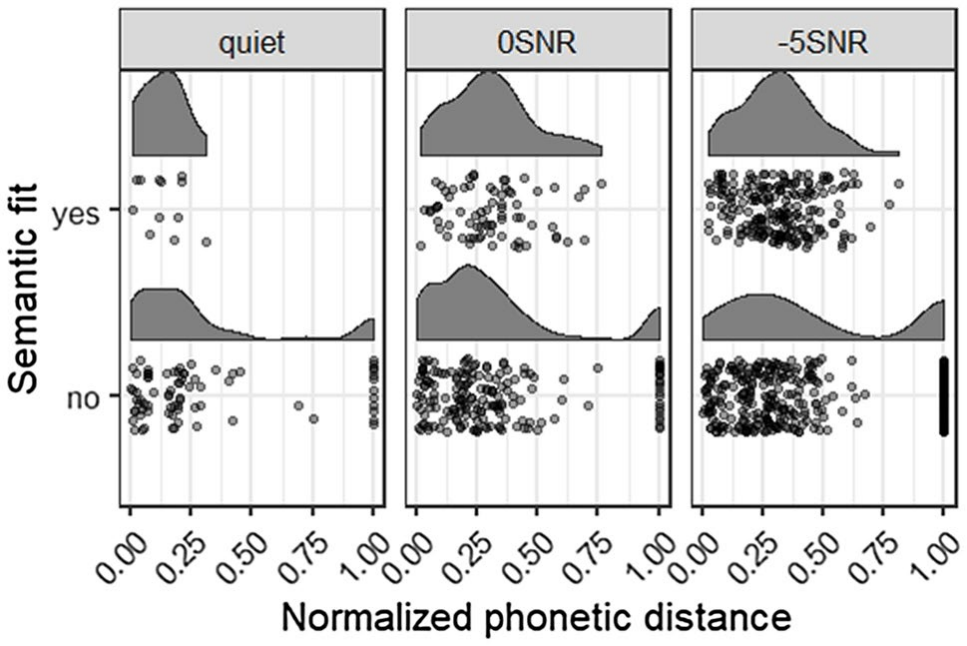
This figure shows the wrong responses that semantically fit or did not fit the sentence, plotted with the normalized phonetic distance, in each of the three noise conditions. Lower phonetic distance means more similar to the target item. A distance of 1 means an empty response.

We coded the semantic fit and phonetic distance to the target of the wrong responses, to see whether participants relied more on the acoustic signal (low distance) or on the provided context (wrong response fits semantically). This gives more insight in the participants’ strategies and allows us to tease apart whether participants relied on top-down (predictions based on context) or bottom-up (acoustic signal) information. Figure 3 presents the normalized phonetic distance and semantic fit for the wrong responses in each of the three noise conditions. Lower normalized phonetic distance scores mean that the participant’s response sounded more similar to the target word. Responses with a distance score of 1 were empty responses. Figure 3 also shows that a majority of the wrong responses in each of the noise conditions, the participant’s response did not fit the sentence semantically (76 vs. 12 for Quiet; 177 vs. 73 for 0 SNR; and 341 vs. 208 for −5 SNR). The peaks of the phonetic distance distributions seem to lie more to the right (meaning larger distance to the target) in the semantically fitting responses, suggesting a trade-off between acoustic fit and semantic fit. Participants made their response based on what they heard at a cost of fitting the semantic context.

### Confidence Ratings

**Figure 4:**
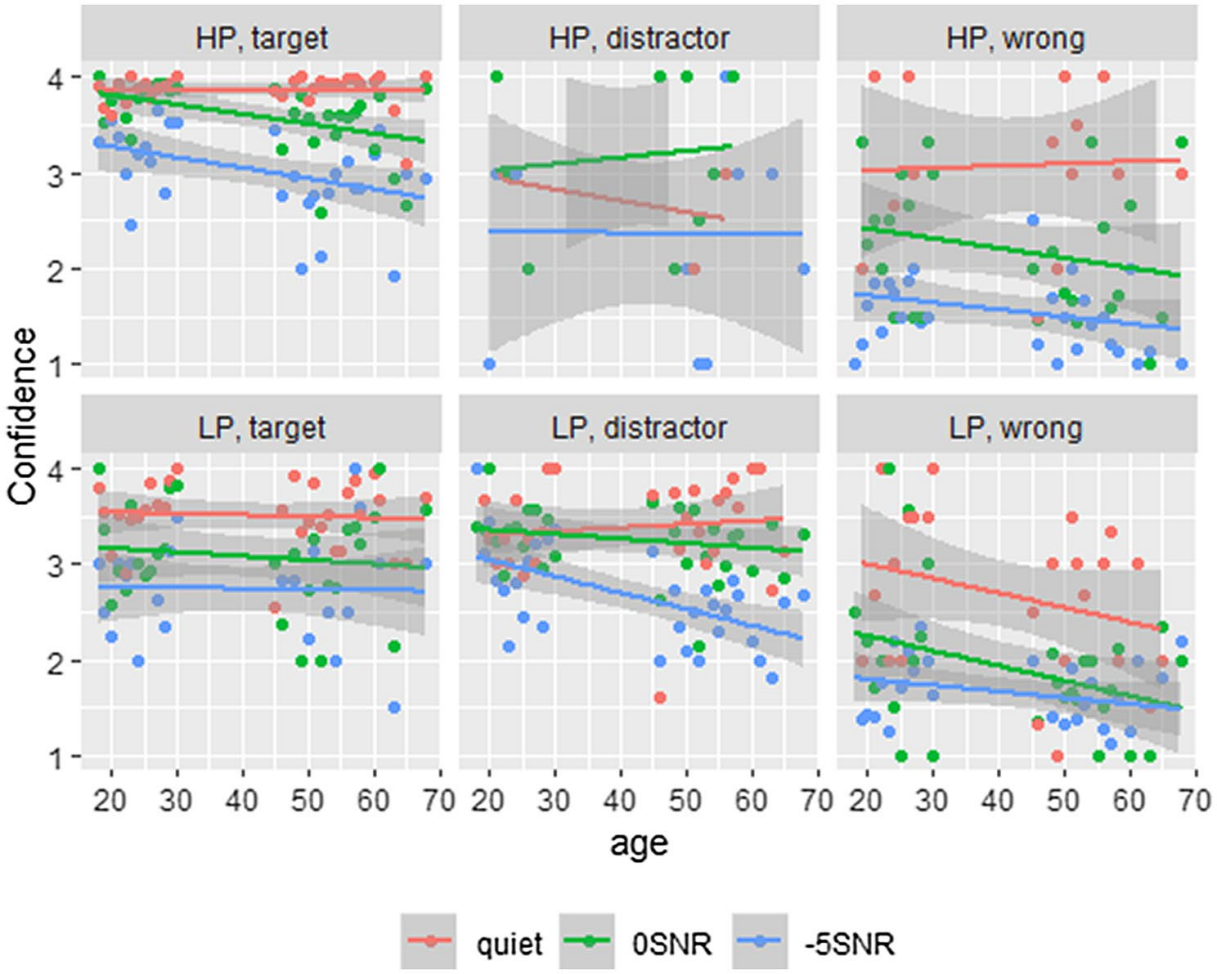
This figure shows the participants’ confidence ratings; split for the predictability conditions, with HP at the top row and LP at the bottom, as well as the three answer types. Age plotted on the x-axis and confidence on the y-axis. Here 1 denotes the lowest confidence and 1 the highest confidence. Different line colors show different noise conditions.

We calculated the mean confidence for each of the three response types, namely, targets (*M*=3.494, *SD*=0.806), distractors (*M*=2.997, *SD*=0.994), and wrong responses (*M*=1.756, *SD*=0.988), finding similar confidence for targets and distractors overall, and lower confidence for wrong responses. We transformed the participants’ confidence responses to a binary variable of low confidence (confidence ratings 1 and 2) and high confidence (confidence ratings 3 and 4). This binary response variable was tested using a GLMM with logistic linking function. Equivalent results are found with ordinal regression analyses. Because of better interpretability, we present the binomial regression here, while results from the ordinal regression can be found in the Supplementary Material. For these analyses, we have taken three subsets of the data: one with the target responses (*N*=4,161), one with the distractor responses (*N*=1,438), and one with the wrong responses (*N*=881). We expected to find different patterns of confidence ratings for these subsets, because in the wrong responses, participants relied mostly on the sentence context, while in the distractor responses, there was some supporting evidence from the acoustic signal as well. As such, we expected participants to be more certain in general of their distractor items, than of their wrong items, as they realized that the wrong items were not presented to them in the speech signal. These analyses will shed light on how confident participants were in the different response types overall. Subsequently, we will turn to the distractor responses in the three noise conditions, as this was the condition most likely to elicit false hearing. Figure 4 presents the participants’ confidence ratings from uncertain (1) to certain (4), split for each of the predictability conditions, noise levels, and response types.

The model for the subset of target responses included fixed effects of Predictability (categorical predictor with two levels using dummy coding and mapping the high-predictability condition on the intercept), Noise, Age, Trial Number, and ContrastVP, as well as the three-way interaction of Predictability, Noise, and Age. All were coded and scaled as before. A by-Participant random intercept was included with random slopes for Noise and Predictability, and a by-Item random intercept with a random slope for Predictability. There was a significant effect of Predictability, with lower confidence in LP vs. HP (*β*=−2.17, *SE*=0.51, *z*=−4.28, *p*<0.001). The model revealed lower confidence in Noise compared to Quiet (*β*=−1.71, *SE*=0.46, *z*=−3.70, *p*<0.001 for 0SNR and *β*=−4.10, *SE*=0.47, *z*=−8.78, *p*<0.001 for −5SNR). The interaction of Noise and Age was significant, with lower confidence for older participants in noise (*β*=−1.07, *SE*=0.36, *z*=−3.02, *p*<0.01 for 0SNR and *β*=−0.85, *SE*=0.34, *z*=−2.52, *p*<0.05 for −5SNR). Finally, the three-way interaction of Predictability, Noise, and Age was significant for the 0SNR condition, with higher confidence ratings with age in LP (*β*=0.99, *SE*=0.41, *z*=2.42, *p*<0.05). The other effects were not significant (all values of *p*>0.08), and all effects can be found in Table 3.

**Table 3:** Model outcomes for the confidence rating analysis (target subset).

	Estimate	*SE*	*z*-value	*value of p*	
Intercept	5.65	0.48	11.69	< 0.001	^***^
Predictability LP	−2.17	0.51	−4.28	< 0.001	^***^
Noise −5SNR	−4.10	0.47	−8.78	< 0.001	^***^
Noise 0SNR	−1.71	0.46	−3.70	< 0.001	^***^
Age	0.15	0.31	0.48	0.63
Trial No	−0.02	0.07	−0.34	0.73
ContrastVP V	0.35	0.20	1.77	0.08
Predictability LP: Noise −5SNR	0.76	0.55	1.38	0.16
Predictability LP: Noise 0 SNR	−0.77	0.49	−1.56	0.12
Predictability LP: Age	0.05	0.36	0.13	0.90
Noise −5SNR: Age	−0.85	0.34	−2.52	< 0.05	^*^
Noise 0SNR: Age	−1.07	0.34	−3.02	< 0.01	^**^
Predictability LP: Noise −5SNR: Age	0.42	0.44	0.97	0.33
Predictability LP: Noise 0SNR: Age	0.99	0.41	2.42	< 0.05	^*^

This table shows the analysis for the subset of target items. The response variable is the participants’ confidence (high or low).

The model for the subset of distractor responses included the same fixed effects as the model on the subset of target responses. A by-Participant random intercept was included, as well as a by-Item random intercept with a random slope of Predictability. Inclusion of other random slopes led to models with a singular fit. The model revealed a significant effect of vowel/plosive contrast (*β*=−0.46, *SE*=0.20, *z*=−2.29, *p*<0.01), suggesting that participants were less confident about their answers on items that had a vowel contrast, rather than those with a plosive contrast. Additionally, there was a significant effect of Trial Number, where participants are less confident in later trials (*β*=−0.19, *SE*=0.08, *z*=−2.43, *p*<0.01). The other effects were not significant (all values of *p*>0.40). All effects can be seen in Table 4.

**Table 4:** Model outcomes for the confidence rating analysis (distractor subset).

	Estimate	*SE*	*z*-value	*value of p*	
Intercept	1.73	2.77	0.62	0.53	^*^
Predictability LP	0.31	2.78	0.11	0.91
Noise −5SNR	−1.51	2.90	−0.52	0.60
Noise 0SNR	0.33	2.94	0.11	0.90
Age	−2.16	3.37	−0.64	0.52
Trial No	−0.19	0.08	−2.43	< 0.05
ContrastVP V	−0.46	0.20	−2.29	< 0.05	^*^
Predictability LP: Noise −5SNR	−0.10	2.90	0.04	0.97
Predictability LP: Noise 0 SNR	−0.32	2.96	−0.11	0.91
Predictability LP: Age	2.63	3.38	0.78	0.44
Noise −5SNR: Age	1.02	3.23	0.31	0.75
Noise 0SNR: Age	2.89	4.29	0.67	0.50
Predictability LP: Noise −5SNR: Age	−1.87	3.25	−0.58	0.56
Predictability LP: Noise 0SNR: Age	−3.57	4.30	−0.83	0.41

This table shows the analysis for the subset of distractor items. The response variable is the participants’ confidence (high or low).

The model for the subset of wrong answer items included the same fixed effects as the previous two models, except that this model did not include a three-way interaction, but only an interaction of Predictability and Noise. A by-Participant random intercept was included, as well as a by-Item random intercept. Inclusion of random slopes led to models with a singular fit. The model revealed a significant effect for both noise conditions. In 0SNR noise, participants were less confident than in quiet (*β*=−1.53, *SE*=0.55, *z*=−2.78, *p*<0.01), an effect that was also found for −5SNR noise (*β*=−3.04, *SE*=0.56, *z*=−5.46, *p*<0.001). These findings show that generally confidence ratings reflect the amount of noise that was presented. None of the other effects were significant (all values of *p*>0.20), and all effects are presented in Table 5.

**Table 5:** Model Outcomes for the confidence rating analysis (wrong subset).

	Estimate	*SE*	*z*-value	*value of p*	
Intercept	0.92	0.52	1.77	0.08	^***^
Predictability LP	−0.08	0.58	−0.13	0.90
Noise −5SNR	−3.04	0.56	−5.46	< 0.001
Noise 0SNR	−1.54	0.55	−2.78	< 0.01	^**^
Age	−0.17	0.13	−1.28	0.20
Trial No	−0.07	0.10	−0.71	0.48
ContrastVP V	−0.28	0.25	−1.11	0.27
Predictability LP: Noise −5SNR	0.29	0.64	0.45	0.65
Predictability LP: Noise 0 SNR	−0.42	0.67	−0.62	0.53

This table shows the analysis for the subset of wrong items. The response variable is the participants’ confidence (high or low).

Finally, we want to investigate directly the false hearing effect in the noise conditions, thus focusing on the confidence ratings in mishearings. We take subsets of the data of all distractor items produced in 0SNR (*N*=646), −5SNR (*N*=618), and quiet (*N*=174). Based on previous findings, we expect to find a false hearing effect in the noise conditions, where participants show high confidence in their incorrect responses as these distractor responses were supported by the sentence context. We expect to find an effect of age, so that older participants are more confident of their response than younger adults. All outcomes from the three GLMMs are presented in Table 6.

**Table 6:** Model outcomes for the false hearing analysis.

	Quiet subset				0SNR subset					−5SNR subset				
	Estimate	*SE*	*z*-value	*value of p*	Estimate	*SE*	*z*-value	*value of p*		Estimate	*SE*	*z*-value	*value of p*	
Intercept	0.95	1.77	0.54	0.59	1.89	0.93	2.03	< 0.05	^*^	0.29	0.84	0.35	0.73	
Predictability LP	1.47	1.79	0.82	0.41	0.36	0.92	0.40	0.69		0.22	0.84	0.26	0.79
Age	0.50	0.35	1.43	0.15	−0.17	0.15	−1.22	0.22	^**^	−0.44	0.14	−2.99	< 0.01	^**^
Trial No	−0.36	0.29	−1.23	0.22	−0.36	0.13	−2.85	< 0.01		< 0.001	0.11	0.01	0.99
ContrastVP V	0.34	0.58	0.59	0.55	−0.91	0.28	−3.29	< 0.01	^**^	−0.14	0.27	−0.53	0.59

This table shows the analysis for the subset of distractor items in quiet, 0SNR, and −5SNR. The response variable is the participants’ confidence (high or low).

The model on the subset of 0SNR trials included fixed effects of Predictability, Age, Trial Number, and ContrastVP (all coded and scaled as before). The model also included random intercepts for Subject and Item (random slopes led to non-convergence or singular fit). The model showed significantly lower confidence as the trials went on (*β*=−0.35, *SE*=0.12, *z*=−2.85, *p*<0.01). Additionally, confidence ratings were significantly lower for items with a vowel contrast compared to items with a plosive contrast (*β*=−0.91, *SE*=0.28, *z*=−3.29, *p*<0.01). The other effects were not significant (all values of *p*>0.22).

The model on the subset of −5SNR trials consisted of the same fixed and random effects as the 0SNR model. We find only a significant effect of Age, where older participants are less confident of their responses than younger adults (*β*=−0.44, *SE*=0.15, *z*=−2.99, *p*<0.01). This is the opposite of what we would expect for false hearing based on previous findings (Rogers et al., 2012; Failes et al., 2020), where older participants are *more* confident of their responses. None of the other effects were significant (all values of *p*>0.31).

The model on the quiet subset of the data again included the same fixed and random effects as the previous two models. None of the effects were significant (all values of *p*>0.15). These models together show no evidence for false hearing in our data, although mishearings were frequent.

## Discussion

In the present study, we investigated word recognition in background noise in younger and older adults, analyzing to what extent listeners rely on the acoustic speech signal or on top-down predictions made based on the sentence context. In our experiment, participants typed in the last word of the sentence that was played in quiet or embedded in background noise at 0SNR and −5SNR. Additionally, participants rated their confidence in giving the correct answer. The results showed that in quiet listening conditions, listeners of all ages and in both high- and low-predictive contexts, mainly make use of the information in the acoustic speech signal. However, they turn more to the sentence context than the acoustic signal as a guide when there is some level of background noise. This effect is stronger for older adults than for younger adults, and it is more pronounced in higher levels of background noise, in line with our hypotheses. Generally, we find that words with a vowel contrast are easier to recognize than words with a plosive contrast, a benefit that lessens with age, presumably due to floor effects. With regard to the confidence ratings, we generally find lower confidence ratings that reflect more difficult listening conditions and incorrect answers. Words with vowels get lower confidence ratings when the response is incorrect compared to items with a plosive contrast. In none of the conditions in our experiment do, we find a false hearing effect where participants rate their incorrect responses with higher confidence, even though mishearings were very common.

### Sound Contrast

We carefully controlled the phonetic contrasts of our minimal pairs to investigate how the sound difference of the minimal pair might have an effect on recognition scores. Our pairs differed either in a plosive (place of articulation) or in a vowel (tense/lax). We expected that the items differing in the plosive were more difficult to recognize correctly than the items differing in a vowel. Plosives consist of a relatively short sound, especially compared to vowels that have a longer duration and greater amplitude. Thus, plosives are more likely to get lost in the noise, in which case the listener would make use of the provided sentence context and report having heard the distractor item. This expectation was confirmed by our data. Other studies that looked at a wider range of plosives and vowels also found that, especially in more difficult listening situations, vowels led to easier recognition than plosives (Fu et al., 1998; Cutler et al., 2004).

Our results showed an interaction with age: The facilitative effect of a vowel contrast over a plosive contrast decreased as participants were older. The direction of this interaction is unexpected at first glance, as we had hypothesized that older adults would have increased difficulty identifying plosives, as for these sounds the higher frequencies are more informative than for vowels (Edwards, 1981; Alwan et al., 2011). These high frequencies are lost first in age-related hearing loss (Gates and Mills, 2005). We believe however that the observed interaction is the result of a floor effect: Older adults have a lot of trouble understanding the plosive correctly in noisy conditions, and almost always mistake the distractor for the target item in this condition. As there is already a substantial number of distractor responses for plosives even in the quiet condition, the decline in noise cannot be as steep as the one observed for vowels, for which comprehension is a lot better in quiet. Another possible explanation for the interaction effect might be that the older adults might have had age-induced hearing loss, in which they struggle, among other things, to discriminate spectral transitions in noise (Tun et al., 2012). This difference in mishearing between plosives vs. vowels suggests that even minor changes in how well the acoustic signal can be perceived affects the probability distribution of the bottom-up information and can lead to a more dominant top-down probability, as predicted by the noisy channel model. If, as suggested by Rogers et al. (2012), mishearing is caused by general deficits in cognitive control, we would expect to find no differences between the two sound types.

When looking at the confidence ratings, we find an effect of ContrastVP in the subset of distractor responses. This suggests that participants were less confident of their response if the target word was part of a minimal pair containing a vowel contrast, than when the word came from a pair with a plosive contrast. Most distractor responses were made in the low-predictability condition, where the sentence context supported the distractor word, while the acoustic information did not. We also found that in the low-predictability condition, words from a pair differing in the vowel generally were easier to identify correctly (participants responding with the target word more often than the distractor). When participants responded incorrectly (with the distractor rather than the target), they were less confident of this, suggesting that they were more aware that they misheard the word than they were for plosive contrasts.

We did not choose our sound contrasts with any models of speech perception in mind. In hindsight, our contrasts might not all be processed in the same way. For example, studies suggest that the coronal place of articulation for consonants is not specified and that it can vary freely for coronal consonants (Friedrich et al., 2006; Lahiri and Reetz, 2010; Roberts et al., 2013). We used the coronal sounds /t/ and /d/ in our consonant minimal pairs, contrasted with other plosives differing in place of articulation. Testing whether these sounds led to more distractor responses due to unspecified coronal place of articulation is outside the scope of this article, but would be an interesting question for future research.

### Bottom-Up and Top-Down Processes

This study investigated how bottom-up auditory processes and top-down predictive processes interact in speech comprehension, in particular in noisy conditions and while looking at differences between younger and older adults. In the high-predictability condition of our experiment, we found an effect of noise, so that there were more distractor responses in the conditions with background noise compared to quiet. This effect was small, and most responses were in fact correct, suggesting a ceiling effect, in particular in quiet. In our paradigm, we presented the sentence context on the screen in written form, which will have led to these ceiling effects. Both the information provided by the speech signal and the information provided by the sentence context pointed to the target word. Participants could thus use information from both sources to recognize the correct word, there was no conflict between them. Especially in the quiet condition, there was no expectation that participants would identify the word incorrectly. The fact that we found this ceiling effect shows that our participants were paying attention to the task. The lack of an age effect in the high-predictability condition regarding the number of distractor responses even in noise shows that older adults can make up for difficult listening conditions by making use of the predictability of the message (Wingfield et al., 2005). As this is arguably the most frequent situation in normal language comprehension – i.e., words fit the context – this is a helpful strategy in everyday listening.

We found different results in the low-predictability condition, where the participants’ answers depended greatly on the condition the items were presented in. In the low-predictability condition, the information provided by the acoustic signal is contradicted by the information given by the sentence context, as both point to different lexical items. On the one hand, the word supported by the context is also partially supported by the speech signal. Because we used minimal pairs, these two words only differed in one single phonetic feature. On the other hand, the word supported by the information from the speech signal is not supported by the sentence context at all. In the quiet condition, participants identified the sentence-final word for the most part correctly. In conditions with background noise, however, participants do rely more on the sentence context to guide word recognition, as shown by the shift to a large proportion of distractor answers. The increased rates of mishearing in noise are observed for both younger and older adults, but the effect is substantially stronger for older adults. This is in line with previous work that has shown that older adults tend to rely more heavily on the sentence context (Hutchinson, 1989; Pichora-Fuller et al., 1995; Sommers and Danielson, 1999; Dubno et al., 2000). Due to the presence of noise, it is more difficult to identify all the sounds in the speech signal, and here listeners turn to the other source of information they have available. This was an expected finding, as in previous studies, also younger adults do rely more on context when listening conditions get harder (Hutchinson, 1989; Dubno et al., 2000; Pichora-Fuller, 2008). We also observed a significant learning effect in our data: As the trials in a block proceed, participants are slightly more likely to get the target item correct. This holds for participants irrespective of age. One possible explanation for this is that they became aware of the manipulation and the fact that the context could be misleading, thus paying more attention to the sound signal than they did before. Listeners have been found to be able to re-weight cues based on their statistical properties (Bushong and Jaeger, 2019). It also shows that older adults are able to adapt to the task, unlike in Rogers et al. (2012). In the present study, they learned over the course of the experiment that context might be misleading and weighing the acoustic information more than the top-down predictions. Adaption suggests that older participants are behaving rationally when showing false hearing.

Analyses of semantic fit and phonetic distance to the target word show that the majority of the wrong responses did not fit the sentence semantically, while distances were smaller in the semantically incongruent responses. This suggests that participants did try to rely on the acoustic signal rather than the provided context, somewhat against our expectations. It might be the case that they had noticed the sometimes misleading sentence context and relied less on this information. Even though we already find high rates of mishearing in our study, it is likely that this underestimates the amount of mishearing that would occur for these materials in a more naturalistic setting. Participants were aware of the possible semantic mismatches in the presented audio and sentence context, and our analyses show that participants in fact paid considerable attention to the acoustic signal rather than the sentence context.

According to the noisy channel model (Levy, 2008), information from both sources are combined rationally. However, older adults have been found to rely more on top-down predictive processes than younger adults, which can lead to mishearing in cases when the target is not predicted by the context. A study by Gibson et al. (2013) showed that human language processing relies on rational statistical inference in a noisy channel. Their model predicts that semantic cues should point the interpretation in the direction of plausible meanings even when the observed utterance differs from this meaning, that these non-literal interpretations increase in noisier communicative situation, and decrease when the semantically anomalous meanings are more likely to be communicated. The findings from the present study are in line with the predictions based on the model by Gibson et al.: In more adverse listening conditions, i.e., the conditions with more background noise, listeners rely more on the sentence context to compensate for the difficulties introduced in auditory processing. In these cases, listeners respond that they heard a word that fits the sentence context (plausible meaning), rather than the word that was actually presented to them (implausible meaning). There is contextual information, as well as some sensory information (the shared sounds of the presented word, as these words form a minimal pair) to support the word favored by the sentence context. However, following Gibson et al.’s final prediction, over the course of the experiment participants noticed that the sentence context is not always reliable and showed a learning effect. They came to expect low-predictability sentence-final items, which led to less mishearing.

Rationally combining bottom-up and top-down information in speech comprehension is sensible, in particular in cases of a noisy channel, where the bottom-up signal is partially obscured. However, when the top-down predictions form a mismatch with the information being transferred in the signal, a too strong reliance on top-down processes can lead to problems in communication, in the form of mishearing. These are a side effect of rationally combining bottom-up and top-down information.

### False Hearing

We also tested the replicability of the false hearing effect in German that was reported for English in previous literature (Rogers et al., 2012; Sommers et al., 2015; Failes et al., 2020). This effect generally has been found to be stronger for older adults than younger adults. Unlike previous studies and against our expectations, we do not find an age effect for false hearing in our study, i.e., while there was a substantial amount of mishearing, older participants were not more confident about their responses than younger participants. We also do not find an effect of age on confidence in distractor responses overall. While Rogers et al. (2012) do report a smaller false hearing effect in the condition with loud noise compared to the condition with moderate noise, they do still find a false hearing effect. In the present study, we do not find a significant effect of age at all for the 0 SNR subset, while in −5 SNR the effect is opposite to our expectations: With age, participants become less confident. One possible explanation for this failure to replicate the false hearing effect in noise is the age of the participants: The participants in previous studies were generally older than those in the present study, and thus perhaps more likely to show the false hearing effect due to age-related cognitive declines on top of the effects of mishearing predicted by the noisy channel model. Instead of false hearing, we find that our participants’ confidence ratings reflect the difficulty of the listening condition: They tended to be lower in noisy conditions and in low-predictability sentences.

### Limitations

One of the limitations of this study is that, due to collecting the data via the web, we were not able to collect hearing thresholds of our participants nor were we able to carefully control the sound levels at which the stimuli were presented. We excluded older participants with a large number of incorrect responses in quiet, so that we make sure that the performance in that condition was equated to younger adults. In hindsight, there is another option for controlling hearing levels among our participants. We could have used an alternative control condition where no context cues are available. These stimuli could have been filler sentences in which participants could only rely on the speech signal to make their response. In this way, auditory performance could be equated among our groups of younger and older adults. Peelle et al. (2016) showed that for intelligibility ratings, online testing is a feasible method to replace laboratory testing as it gave comparable results as testing in the laboratory. This suggests that careful control of participants’ listening conditions and software used like in laboratory settings is not necessary to obtain reliable results. Additionally, previous studies have equated overall audibility for older and younger adults using individual speech recognition thresholds, and still found larger false hearing effects for older adults, suggesting it is not directly caused by differences in hearing acuity (Rogers et al., 2012; Sommers et al., 2015; Failes et al., 2020).

We constructed the items in our low-predictability condition by swapping the two words from the minimal pairs we had selected. It should be noted that this lead to sentences that, while unpredictable, also were implausible. In fact, in the low-predictability condition, the sentences provided a context that was strongly biased for the distractor word. This could have led to larger amounts of mishearing compared to when we would have used sentences that were unpredictable but plausible, in particular for older adults who tend to rely more on context. Due to the strong bias for the distractor and the implausibility of the target word, relying on the context would strongly favor the distractor response. Other studies investigating false hearing using sentences varied in whether their low-predictability items were plausible or not. Sommers et al. (2015) used unpredictable sentences that were still meaningful (LP: *The shepherd watched his sheath*), but Failes et al. (2020) had implausible items. They constructed their unpredictable items by changing one phoneme in the sentence-final target word in the predictable item (HP: *She put the toys in the box*; LP: *She put the toys in the fox*). Both these studies found a larger false hearing effect for older adults, and therefore, this effect seems to be independent of the plausibility of the low-predictability items. It therefore seems unlikely that our lack of an effect can be explained by having used implausible sentences. The false hearing effect has also been found using a word priming paradigm (Rogers et al., 2012; Rogers, 2017), which suggests that the effect does not depend on the use of a particular paradigm.

Another limitation of the present study is the age of our older adults, which is relatively young. Our oldest participant was 68years old, and mean age of the older group was 53. Compare this to the ages of the older participants in Failes et al. (2020), which ranged from 65 to 81, with a mean of 71. This might explain the lack of an age-related false hearing effect in the present study. For our sample, we find that rational processes better explain our results of differences between vowel contrasts and plosive contrasts, but of course it could be the case that in an older sample, general cognitive decline plays a part as well (Rogers et al., 2012).

The present results are based on a restricted set of minimal pairs, namely, pairs of plosives only differing in place of articulation, and tense vs. lax vowels, and were tested in multi-speaker babble noise. More research is needed to investigate how these findings generalize to other sound combinations and other types of noise. Future studies could also test at different SNR levels, to prevent in particular the floor effects we found in the plosives as noise, as this can shed light on the true nature of the interaction effect of age and sound contrasts in noise. Currently, the noisy channel model does not incorporate metacognitive measures like confidence ratings. Confidence could be formulated in terms of the probability distribution between different lexical candidates. If, on the one hand, the probability of one candidate is a lot higher than that of another candidate, high confidence in the response should be reported. On the other hand, if the probabilities of different candidates are more similar, the confidence rating should be lower. The exact modeling of false hearing based on confidence ratings in the noisy channel model can be explored in future research.

## Conclusion

Previous studies have investigated the mishearing effect, where listeners understand a word different from the one that was spoken. These effects are particularly prevalent in situations where the speech signal is noisy, and the word that is actually understood fits well with the semantic context, indicating that top-down predictability of the word may have overpowered the bottom-up auditory signal. Previously, this effect has been attributed to general deficits in cognitive control, in particular inhibition (Rogers et al., 2012; Sommers et al., 2015; Failes et al., 2020).

In the present article, we argue that the effect is a natural consequence of rational language processing in noise and thus does not require to be attributed to deficits in cognitive control. To test this idea, we designed a study which carefully controls the way in which the target and the distractor words differ from one another. Specifically, we constructed target-distractor pairs which only differed in the articulatory position in a plosive, and another set of target-distractor pairs that differed only in vowel quality. We conducted an online study in German, in which participants listened to sentences in quiet and two levels of background babble noise, and reported the sentence-final word they heard, as well as rated their confidence in this response. Our findings show that participants accurately report the actually spoken word in quiet listening conditions, but that they rely more on sentence context in the presence of background noise (both babble and white noise), leading to incorrect responses in particular in the low-predictability condition. While listeners thus do profit from high-predictability in noise (as they do correctly understand the words in this condition), they also suffered the downside of mishearing in the low-predictability condition. The mishearing effect was found to be larger in older adults compared to younger adults, replicating previous findings. We explain this within the noisy channel account in terms of increased language experience of older adults, possibly compounded with first experiences of hearing loss.

For our critical phonetic manipulation, we found that stimuli pairs with a vowel contrast were generally easier to identify correctly than pairs with a plosive contrast, although this benefit lessened with age. These different effects for vowels vs. plosives suggest that mishearing depends on the quality of the acoustic signal, rather than general deficits in cognitive control or inhibition. We also find a learning effect that suggests that participants of all ages were able to adapt to the task. We think that this finding also underscores the rational account and is not consistent with an account that relates age differences to a difference in cognitive control. Our findings also add to the literature by replicating the earlier mishearing effects in a different language, German.

Earlier work had however also reported an effect of false hearing, meaning that participants are very confident of their answer even though it is in fact incorrect (Rogers et al., 2012; Sommers et al., 2015; Failes et al., 2020). In particular, the false hearing effect was found to be increased in older adults. While our experiment was also set up to assess false hearing, we did not find any significant effects of false hearing in the older participants compared to the younger ones. Instead, confidence was related to the level of noise.

## Data Availability Statement

The raw data supporting the conclusions of this article will be made available by the authors, without undue reservation.

## Ethics Statement

The studies involving human participants were reviewed and approved by Deutsche Gesellschaft für Sprachwissenschaft (Ethics Committee of the German Linguistic Society). The patients/participants provided their written informed consent to participate in this study.

## Author Contributions

MV, VD and JK were involved in planning and designing of the study. MV analyzed the data and wrote all parts of the manuscript. VD and JK made suggestions to the framing, structuring, and presentation of findings as well as on the interpretation of findings. All authors contributed to the article and approved the submitted version.

## Conflict of Interest

The authors declare that the research was conducted in the absence of any commercial or financial relationships that could be construed as a potential conflict of interest.

## Publisher’s Note

All claims expressed in this article are solely those of the authors and do not necessarily represent those of their affiliated organizations, or those of the publisher, the editors and the reviewers. Any product that may be evaluated in this article, or claim that may be made by its manufacturer, is not guaranteed or endorsed by the publisher.

## Footnotes

^1^ http://bbcsfx.acropolis.org.uk/
