# Supplementary material for: Mishearing as a Side Effect of Rational Language Comprehension in Noise
Source: Front Psychol. 2021 Sep 6;12:679278. doi: 10.3389/fpsyg.2021.679278 (PMC8450506; doi:10.3389/fpsyg.2021.679278)
Supplement: Supplementary file 1 [file Data_Sheet_1.PDF]

frontiers

Supplementary Material

1 Ordinal Regression Confidence Ratings

We conducted ordinal regressions of all analyses involving binarized confidence ratings in the manuscript. We find the same significant effects in the same directions as in the binarized analyses, with some exceptions. In the subset of quiet items, we find a negative effect of trial number, which we did not find before. Participants become less confident of their distractor responses as the experimental blocks went on. In the subset of wrong responses, we now find a significant effect of age, with lower confidence ratings for older participants. Finally, in the subset of target responses we find two new effects: We find a significant interaction of predictability and noise at -5SNR, leading to higher confidence ratings. Beyond the positive effect of the three-way interaction of predictability, age, and noise at 0SNR, we now also find a positive effect for this three-way interaction for noise at -5SNR.

The ordinal regressions were implemented in the ordinal package (Christensen, 2015) in R (R Development Core Team). We ran cumulative link mixed models (CLMM) that included the same fixed and random effects as the GLMMs with the binarized confidence ratings, which will be repeated below. First, we ran separate models on the three different response types (target, distractor, and wrong responses), and secondly, we ran separate models on the three subsets of distractor responses for the different noise types (quiet, 0SNR, -5SNR).

The model for the subset of target responses included fixed effects of Predictability (categorical predictor with two levels using dummy coding, mapping the High Predictability condition on the intercept), Noise (categorical predictor with three levels, mapping Quiet to the intercept), Age (scaled), Trial Number (scaled), ContrastVP (categorical predictor with two levels using dummy coding, mapping Plosive to the intercept), as well as the three-way interaction of Predictability, Noise, and Age. A by-Participant random intercept was included with random slopes for Noise and Predictability, and a by-Item random intercept with a random slope for Predictability. The model revealed a significant effect of Predictability, with lower confidence in LP versus HP (β = -2.50, SE = 0.24, z = -10.59, p < .001). The model revealed lower confidence in Noise compared to Quiet (β = -2.03, SE = 0.23, z = -8.86, p < .001 for 0SNR, and β = -4.26, SE = 0.26, z = -8.86, p < .001 for -5SNR). We find a significant interaction for Predictability and Noise, but only for the -5SNR condition, with higher confidence ratings in LP items (β = 1.21, SE = 0.28, z = 4.33, p < .001). The interaction of Noise and Age was significant, with lower confidence for older participants in noise (β = -0.75, SE = 0.20, z = -3.69, p < .001 for 0SNR, and β = -0.80, SE = 0.22, z = -3.56, p < .001 for -5SNR). Finally, the three-way interaction of Predictability, Noise, and Age was significant, with higher confidence ratings with age in LP in noise (β = 0.75, SE = 0.22, z = 3.46, p < .001 for 0SNR, β = 0.84, SE = 0.26, z = 3.30, p < .001 for -5SNR). The other effects were not significant (all p-values > .06), all effects can be found in Table 1.

The model for the subset of distractor responses included the same fixed effects as the model on the subset of target responses. A by-Participant random intercept was included, as well as a by-Item random intercept with a random slope of Predictability. The model revealed a significant effect of Vowel/Plosive contrast (β = -0.48, SE = 0.18, z = -2.71, p < .01), suggesting that participants were less confident about their answers on items that had a vowel contrast, rather than those with a plosive

contrast. Additionally, there was a significant effect of Trial Number, where participants are less confident in later trials (β = -0.15, *SE* = 0.06, *z* = -2.61, *p* < .01). The other effects were not significant (all *p*-values > .31). All effects can be seen in Table 2.

The model for the subset of wrong answer items included the same fixed effects as the previous two models, except that this model did not include a three-way interaction, but only an interaction of Predictability and Noise. A by-Participant random intercept was included, as well as a by-Item random intercept. The model revealed a significant effect for both noise conditions, with lower confidence ratings in noise (β = -1.62, *SE* = 0.47, *z* = -3.47, *p* < .001 for 0SNR, and β = -3.27, *SE* = 0.46, *z* = -7.10, *p* < .001 for -5SNR). Additionally, the model showed a significant effect of Age, with lower confidence ratings for older participants (β = -0.37, *SE* = 0.12, *z* = -3.10, *p* < .01). None of the other effects were significant (all *p*-values > .13), and all effects are presented in Table 3.

Table 3

Model Outcomes for the Confidence Rating Analysis (Target Subset)

	Estimate	*SE*	*z*-value	*p*-value	
Predictability LP	-2.50	0.24	-10.59	<.001	***
Noise 0SNR	-2.03	0.23	-8.86	<.001	***
Noise -5SNR	-4.27	0.26	-16.45	<.001	***
Age	0.15	0.23	0.65	.52	
Trial No	-0.05	0.04	-1.22	.22	
ContrastVP V	0.26	0.14	1.87	.06	.
Predictability LP : Noise 0 SNR	-0.02	0.24	-0.07	.94	
**Predictability LP : Noise -5SNR**	**1.21**	**0.28**	**4.33**	**<.001**	***
Predictability LP : Age	-0.33	0.20	-1.65	.10	.
Noise 0SNR : Age	-0.75	0.20	-3.69	<.001	***
Noise -5SNR : Age	-0.80	0.22	-3.56	<.001	***
Predictability LP : Noise 0SNR : Age	0.75	0.22	3.46	<.001	***
**Predictability LP : Noise -5SNR : Age**	**0.84**	**0.26**	**3.30**	**<.001**	***

Note. This table shows the analysis for the subset of target items. The response variable is the participants' confidence (high or low). Rows in bold denote new effects compared to the binomial analyses in the manuscript.

Table 2

Model Outcomes for the Confidence Rating Analysis (Distractor Subset)

	Estimate	*SE*	*z*-value	*p*-value	
Predictability LP	1.08	1.06	1.03	.31	
Noise 0SNR	1.06	1.22	0.87	.38	
Noise -5SNR	-0.74	1.19	-0.63	.53	
Age	-0.53	1.26	-0.42	.68	
Trial No	-0.15	0.06	-2.61	<.01	**
ContrastVP V	-0.48	0.18	-2.71	< .01	**
Predictability LP : Noise 0SNR	-1.05	1.24	-0.85	.40	
Predictability LP : Noise -5SNR	-0.57	1.20	-0.47	.64	
Predictability LP : Age	0.64	1.27	0.51	.61	
Noise -5SNR : Age	0.90	1.65	0.54	.59	
Noise 0SNR : Age	0.25	1.38	0.18	.86	
Predictability LP : Noise 0SNR : Age	-1.27	1.67	-0.76	.45	
Predictability LP : Noise -5SNR : Age	-0.85	1.40	-0.60	.55	

Note. This table shows the analysis for the subset of distractor items. The response variable is the participants' confidence (high or low).

Table 3

Model Outcomes for the Confidence Rating Analysis (Wrong Subset)

	Estimate	*SE*	*z*-value	*p*-value	
Predictability LP	-0.62	0.49	-1.25	.21	
Noise 0SNR	-1.62	0.47	-3.47	<.001	***
Noise -5SNR	-1.54	0.55	-2.78	<.001	***
**Age**	**-0.37**	**0.12**	**-3.10**	**<.01**	**
Trial No	0.01	0.08	0.07	.94	
ContrastVP V	-0.09	0.21	-0.41	.68	
Predictability LP : Noise 0SNR	0.18	0.56	0.32	.75	
Predictability LP : Noise -5SNR	0.81	0.53	1.52	.13	

Note. This table shows the analysis for the subset of wrong items. The response variable is the participants' confidence (high or low). Rows in bold denote new effects compared to the binomial analyses in the manuscript.

The model on the subset of 0SNR trials included fixed effects of Predictability, Age, Trial Number, and ContrastVP (all coded and scaled as before). The model also included random intercepts for Subject and Item. The model showed significantly lower confidence as the trials went on (β = -0.24, *SE* = 0.10, *z* = -2.49, *p* < .05). Additionally, confidence ratings were significantly lower for items with a Vowel contrast compared to items with a Plosive contrast (β = -0.67, *SE* = 0.24, *z* = -2.77, *p* < .01). The other effects were not significant (all *p*-values > .17).

The model on the subset of -5SNR trials consisted of the same fixed and random effects as the 0SNR model. We find only a significant effect of Age, where older participants are less confident of their responses than younger adults (β = -0.51, *SE* = 0.16, *z* = -3.18, *p* < .01). None of the other effects were significant (all *p*-values > .19).

The model on the quiet subset of the data again included the same fixed and random effects as the previous two models. We only find a significant effect of Trial (β = -0.45, *SE* = 0.20, *z* = -2.24, *p* < .05). Other effects were not significant (all *p*-values > .16). All outcomes from these three GLMMs are presented in Table 4.

Table 4
Model Outcomes for the False Hearing Analysis

	Quiet subset					0SNR subset					-5SNR subset				
	Estimate	*SE*	*z*-value	*p*-value		Estimate	*SE*	*z*-value	*p*-value		Estimate	*SE*	*z*-value	*p*-value	
Predictability LP	1.75	1.24	1.42	.16		0.32	0.81	0.39	.70		0.71	0.69	1.02	.31	
Age	0.24	0.25	0.93	.35		-0.22	0.16	-1.38	0.17		-0.51	0.16	-3.18	<.01	**
**Trial No**	**-0.45**	**0.20**	**-2.24**	**<.05**	*	-0.24	0.10	-2.49	<.05	*	0.03	0.09	0.28	.78	
ContrastVPV	-0.31	0.37	-0.84	.40		-0.67	0.24	-2.77	<.01	**	-0.31	0.24	-1.32	.19	

Note. This table shows the analysis for the subset of distractor items in quiet, 0SNR, and -5SNR. The response variable is the participants' confidence (high or low). Rows in bold denote new effects compared to the binomial analyses in the manuscript.

The outcomes from this ordinal regression do not change our conclusions based on the binarized confidence ratings reported in the main manuscript. If anything, the additional significant effect of trial number in the quiet subset suggest that participants learned to trust their distractor responses less, while the negative effect of age in the wrong responses shows that older adults were less confident of their responses than younger adults. This is the opposite of what we would expect for false hearing. We do find positive effects on confidence ratings in the subset of target responses, but as these are correct responses, they do not signify false hearing.

References

Christensen, R. H. B. (2015). ordinal—regression models for ordinal data. R package version, 28, 2015.

R Development Core Team. (2020). R: A Language and Environment for Statistical Computing. R Foundation for Statistical Computing, Vienna, Austria. https://wwwR-project.org/
